# HLA-E gene polymorphism associates with ankylosing spondylitis in Sardinia

**DOI:** 10.1186/ar2860

**Published:** 2009-11-13

**Authors:** Fabiana Paladini, Francesca Belfiore, Elisa Cocco, Carlo Carcassi, Alberto Cauli, Alessandra Vacca, Maria Teresa Fiorillo, Alessandro Mathieu, Isabella Cascino, Rosa Sorrentino

**Affiliations:** 1 Department of Cell Biology and Development, University "La Sapienza", via dei Sardi, 70, 00185 Roma, Italy; 2 Cell Biology Institute, CNR, via E. Ramarini, 00016 Monterotondo Scalo, Roma, Italy; 3 Chair of Medical genetics, Department of Internal Medicine, University of Cagliari, SS 554 09042 Monserrato, Cagliari, Italy; 4 Chair of Rheumatology II, Department of Medical Sciences, University of Cagliari, SS 554 09042 Monserrato, Cagliari, Italy; 5 Fondazione Pasteur-Cenci Bolognetti, University "La Sapienza", Pz. Aldo Moro 5, 00185 Roma, Italy

## Abstract

**Introduction:**

Ankylosing spondylitis (AS) is a severe, chronic inflammatory disease strongly associated with HLA-B27. The presence of additional HLA risk factors has been suggested by several studies. The aim of the current study is to assess the occurrence of an additional HLA susceptibility locus in the region between HLA-E and HLA-C in the Sardinian population.

**Methods:**

200 random controls, 120 patients with AS and 175 HLA-B27 positive controls were genotyped for six single nucleotide polymorphisms (SNPs) spanning the HLA region between HLA-E and HLA-C loci previously shown to harbour an additional susceptibility locus for AS. Allele, genotype and haplotype frequencies were compared.

**Results:**

The data confirm our previous finding of a significant increase in patients with AS of allele A at SNP rs1264457 encoding for an Arg at the functional HLA-E polymorphism (Arg^128^/Gly^128^). This was due to a remarkable increase in the frequency of genotype A/A in patients vs HLA-B27-matched controls (51% vs 29%; *P *for trend: 5 × 10^-5^). Genotype distribution of three other SNPs mapping in genes (GNL1, PRR3 and ABCF-1) close to HLA-E and showing high LD with it, was also significantly skewed. Accordingly, haplotype distribution was also remarkably different. The frequency of the haplotype AAGA, is 42% in random controls, increases to 53% in the HLA-B27-positive controls, and reaches 68% in patients with AS (*P *values: 2 × 10^-11 ^vs random and 3 × 10^-4 ^vs HLA-B27 controls).

**Conclusions:**

There is a strong association between the presence of a haplotype in genes mapping between HLA-E and HLA-C and AS due to an increase of homozygous markers in patients. The strongest association however, is with the HLA-E functional polymorphism rs1264457. Since HLA-E is the ligand for the NKG2A receptor, these data point to the natural killer (NK) activity as possible player in the pathogenesis of AS.

## Introduction

Ankylosing spondylitis (AS) is a severe, chronic inflammatory disease whose strong association with human leukocyte antigen (HLA)-B27 has been known for many years. However, the presence of HLA-B27 far from accounts for the entire genetic load in AS. Other genes within and outside the HLA region have been evoked as additional factors contributing to the disease [1-4]. Although the association of genes outside the HLA region, such as IL-1, IL23R or endoplasmic reticulum aminopeptidase 1 (ERAP1), has been demonstrated, it appears more difficult to single out independent HLA risk factors, due to the strong Linkage Disequilibrium (LD) among genes mapping within the HLA region. The Sardinian is an outlier population and, due to the relative low gene flow from outside populations [5], is particularly suitable for case-control association studies in common complex diseases. In previous work, by comparing HLA-B27 -positive patients with AS and controls, we have reported that the region between HLA-E and major histocompatibility class I polypeptide-related sequence A (MICA) contains genes showing different allele frequencies between the two cohorts, and that there was an excess of homozygous markers in patients [6]. This allowed us to speculate that the co-occurring chromosome was contributing to the disease providing a double dose of the predisposing allele. Interestingly, among the markers analysed, we found that the functional single nucleotide polymorphism (SNP) in the HLA-E gene (rs1264457A/G; determining the amino acid variation Arg128Gly) showed a significantly different genotype distribution in the two cohorts. Here, we analyse a larger cohort of HLA-B27-positive patients (n = 120) and HLA-B27-positive controls (n = 175) for the distribution of rs1264457 and five other SNPs mapping in genes close to the HLA-E locus. This further analysis confirms the strong association between allele A at rs1264457 in the HLA-E gene and AS.

## Materials and methods

In this study, 200 random controls, 120 HLA-B27-positive patients with AS and 175 HLA-B27-matched controls were analysed. Seventy-nine HLA-B27-positive patients and 77 controls (24 of which were typed as B*2709) have been previously described [6]. A further 41 patients with AS and 98 HLA-B27-positive controls (16 of which typed as B*2709) were enrolled in this study. The final subtype distribution was as follow: 108 B*2705 (90%), 6 B*2702 (5%), 4 B*2707 (3%) and 2 B*2713 (2%) in the cohort of patients with AS; and 121 B*2705 (69%), 40 B*2709 (23%), 9 B*2707 (5%) and 5 B*2702 (3%) in the cohort of HLA-B27-positive controls. Population stratification had been controlled by analyzing the distribution of a SNP mapping at the 5' end (rs4988235) of the gene for lactase and no significant difference in genotype distribution between patients and controls was found [7]. DNA samples from patients and controls were collected at the University of Cagliari, Cagliari, Sardinia. The B*2709 haplotype, not associated with AS [8], was not different from B*2705 for the markers analyzed here [6] and therefore B*2709 controls were included in the study. Genotype distribution of all six SNPs was in Hardy-Weinberg equilibrium. All patients were diagnosed according to the modified New York criteria [9]. All subjects gave their informed consent to the study. The study was approved by the local ethics committee.

### PCR

A 20 ng sample of genomic DNA in a volume of 15 ul, containing 20 mM Tris-HCl (pH 8.4), 50 mM KCl, 2.5 mM MgCl_2_, 0.2 mM dNTPs, 5 pmoles each of forward and reverse primer and 0.75 U of Taq Polymerase (Biotaq DNA Polymerase, Bioline (Bioline Ltd, London, UK) was used. The mixture was incubated at 94°C for three minutes followed by 25 to 30 cycles of denaturation at 94°C for 30 seconds, annealing at a range of 55 to 62°C (depending on the analysed marker) for 30 seconds, extension at 72°C for one minute, with a final step of 72°C for 10 minutes.

### SNP typing

Five SNPs mapping in the region encompassing about 100 kb between the HLA-E and HLA-C genes have been analysed: rs2105960 in the member RAS oncogene family pseudogene 1 (RANP1) gene, rs1264457 in the HLA-E gene, rs2074505 in the guanine nucleotide binding protein-like 1 (GNL1) gene, rs2074503 in the proline-rich polypeptide 3 (PRR3) gene, and rs 2269709 and rs1264439 in the ATP-binding cassette, subfamily F (GCN20), member 1 (ABCF-1) gene. SNPs were typed by minisequencing as previously reported [6]. Briefly, the amplified products were treated with 0.5 U of shrimp alkaline phosphatase (SAP; Roche, Gaillard, France) and 2.5 U of exonuclease I (EXO I; BioLabs, Frankfurt am Main, Germany) at 37°C for 120 minutes and at 75°C for 15 minutes for inactivation. For minisequencing reactions, the commercial fluorescent-based minisequencing kit SNaPshot multiplex (Applied Biosystems, Lincoln Centre Drive, Foster City, USA) was used, with 2 pmoles of primer. PCR for single base extension reaction was as follows: initial denaturation at 94°C for three minutes, followed by 50 cycles of primer extension at 96°C for 15 seconds, annealing at 55 to 59°C (depending on the oligonucleotide annealing temperature) for 15 seconds, extension at 60°C for one minute. After the extension and labelling reaction, the unincorporated dNTPs were removed by enzymatic treatment using 0.5 U of SAP at 37°C for 120 minutes and at 75°C for 15 minutes for inactivation. The products were analyzed on the ABI prism 310 Genetic Analysers (Applied Biosystems, Lincoln Centre Drive, Foster City, USA). Genotyping was performed by 310 ABI Prism GeneScan 2.1 software (Applied Biosystems,, Lincoln Centre Drive, Foster City, USA).

Primers were as follows: rs2105960: FOR: AGACGCTTCTGGAAGGAACA; REV: GGCTGTGTCCCATACATTGA; SEQ: ATGGTGGTACTGGAAAAACG. rs1264457: FOR: 5'-GTGGGCGGGACTGACTAAG; REV: 5'-GGTCCTCATTCAGGGTGAGA; SEQ: 5'-TGCGAGCTGGGGCCCGAC. rs2074505: FOR: CACAGTGATGGCTTCACAGGC; REV: 5'-CCTATAGGAAGCCCTCTGAGAC; SEQ: CCCCCGCACCCCACAGGA. rs2074503: FOR: GCACAAGAAACAGCACCAAA; REV: AGTGACACACCCATCCCATC; SEQ: AGGAAGCAAAGATGGGTCCC. rs2269709: FOR: TGCCGCCTCCTTAGTAACTC; REV: TTAGATGCCATTCCGAGGAG; SEQ: TGAACTGGTCAAGATGGAGG. rs1264439: FOR: GATTACAGGCGTGACCCATT; REV: CCCTTTTCCTCTTTCCCTTC; SEQ: CTCACCACCAAGTAGGGAGA.

### Statistical analysis

Two-tailed Fisher's Exact test was used to assess the differences of proportion of polymorphic alleles and association between random controls and HLA-B27 patients, HLA-B*2705 and HLA-B*2709 controls. The Cochrane-Armitage trend test was performed using StatXact7 software (Massachusetts Ave, Cambridge, USA). *P *values less than 0.05 were considered significant. LD was calculated using Haploview software [10]. Hardy-Weinberg equilibrium testing was performed using Pearson's goodness-of-fit chi-squared test with one degree of freedom.

## Results

**Figure 1:**
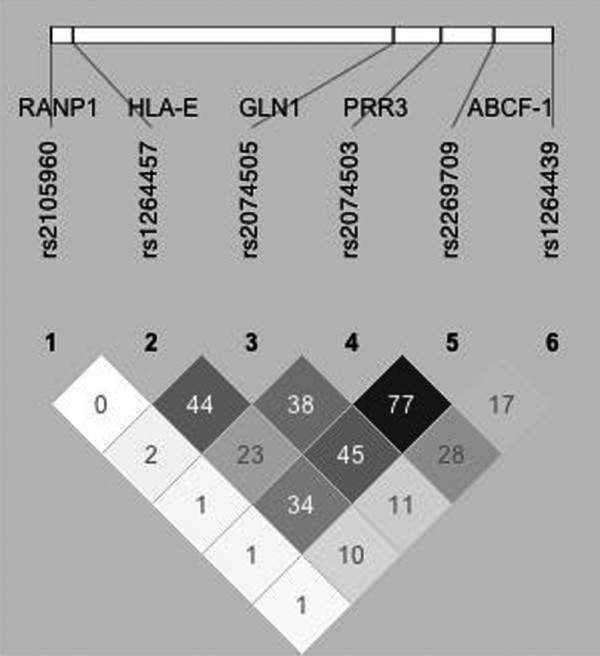
Linkage disequilibrium values among the markers mapping in the region between HLA-E and HLA-C in the Sardinian population. Linkage Disequilibrium r^2 ^values among the six SNPs spanning the region between the HLA-E and HLA-C genes considered in this study. Calculation has been made analyzing two hundreds random controls (400 haplotypes) using haploview software [10]. Color scale reflects the strength of LD among the six markers whose genotype distribution was in Hardy-Weinberg Equilibrium.

We report here allele, genotype and haplotype distribution of six markers spanning the region between HLA-E and HLA-C genes in 175 HLA-B27-positive controls and 120 patients with AS. LD r^2 ^values calculated using 200 random controls, are reported in Figure 1. Marker rs2105960 mapping in the pseudogene RANP1 shows no LD with the others. A central block of LD is evident going from HLA-E rs1264457 to rs2269709 in the ABCF1 gene. Allele frequencies of these six SNPs in HLA-B27-positive patients with AS and controls is reported in Table 1. RANP1 rs 2105960, mapping upstream HLA-E and not in LD with it, shows a similar allele frequency in patients with AS and HLA-B27 controls. According to our previous data [6], HLA-E rs1264457 shows the highest association with AS (*P *= 1 × 10^-4^; odds ratio = 2; 95% confidence interval = 1.4 to 2.9). In parallel with the decrease of the LD, the strength of association of the other markers mapping downstream the HLA-E gene, also decreases. Rs1264439, mapping in the ABCF1 gene, is almost monomorphic and shows no association. As expected, genotype distribution of the HLA-E rs1264457 is significantly different between patients with AS and HLA-B27-positive controls (*P*-trend = 5 × 10^-5^) with a strong increase in genotype AA in patients (51% vs 29%). Genotype AG is present in 60% of B27 controls vs 46% of patients with AS. The other markers in high LD with it, also show significant *P*-trend values which, however, downgrade the more the distance from rs1264457. As expected, haplotype distribution of the four SNPs showing association, rs1264457/rs2074505/rs2074503/rs2269709, is also different between patients and controls (Table 2). HLA-E rs1264457 allele A is a marker of the HLA-B27 haplotype in Sardinia. Accordingly, the haplotype AAGA is significantly over-represented among the HLA-B27-positive controls (53% calculated using the overall cohort of the HLA-B27 controls compared with 42% in random controls. *P *value = 9 × 10^-4^). Haplotype distribution in the HLA-B*2709-positive cohort is reported in Table 2. Although the number of subjects analysed is low (n = 40), it is evident that the distribution is, if any, more similar to that of the AS patients than to the HLA-B27 controls indicating that the same AAGA haplotype is a feature of both the B*2705- and the B*2709-positive cohorts. For this reason, the statistical analysis has always been performed pooling all B27 controls in one single cohort. Due to the homozygous status of the corresponding markers, patients with AS show an even higher frequency of the 'B27 haplotype' (AAGA: 68%). The other frequent haplotype, GGAG, is under-represented in patients compared with the HLA-B27 controls, in which the increase of the haplotype AAGA is counterbalanced by a proportional lower frequency of all the other haplotypes.

**Table 1:** Allele frequencies and Cochrane-Armitage test for trend of genotype distribution in patients with AS and HLA-B27-matched controls in Sardinia

			HLA-B27+ controls n = 175	HLA-B27+ patients with AS n = 120			
						
Gene	SNP	Position on chromosome 6	MAF (%)	MAF (%)	*P*	OR	(95% CI)
RANP1	rs2105960	30561768	245 (75)	154 (69)	ns		
	TT		94 (57)	49 (44)			
	CT		63 (38)	56 (50)			
	CC		9 (5)	7 (6)			
	***P *trend**				**ns**		

HLA-E	rs1264457	30566040	207 (59)	177 (74)	1 × 10^-4^	2	1.4-2.9
							
	AA		51 (29)	61 (51)	Ref		
	AG		105 (60)	55 (46)	1 × 10^-3^	0.44	(0.27-0.72)
	GG		19 (11)	3 (3)	4 × 10^-4^	0.13	(0.04-0.5)
	***P *trend**				**5 × 10^-5^**		

GNL1	rs2074505	30629116	219 (63)	179 (76)	4 × 10^-4^	1.9	1.4-2.8
							
	AA		68 (39)	68 (58)	Ref		
	AG		83 (47)	43 (37)	0.01	0.52	(0.31-0.85)
	GG		24 (14)	6 (5)	4 × 10^-3^	0.25	(0.1-0.65)
	***P *trend**				**4 × 10^-4^**		

PRR3	rs2074503	30638475	247 (76)	203 (85)	9 × 10^-3^	1.8	1.2-2.7
							
	GG		93 (57)	85 (71)	Ref		
	AG		61 (37)	33 (28)	0.05	0.59	(0.35-1)
	AA		10 (6)	2 (1)	0.04	0.22	(0.05-1)
	***P *trend**				**7 × 10^-3^**		

ABCF1	rs2269709	30648869	224 (74)	201 (84)	5 × 10^-3^	1.8	1.2-2.8
							
	AA		82 (54)	83 (69)	Ref		
	AG		60 (39)	35 (29)	0.04	0.58	(0.34-0.99)
	GG		10 (7)	2 (2)	0.03	0.2	(0.04-0-9)
	***P *trend**				**4 × 10^-3^**		

ABCF1	rs1264439	30660481	312 (91)	212 (91)	ns		
							
	GG		142 (83)	97 (84)			
	GT		28 (16)	18 (15)			
	TT		1 (1)	1 (1)			
	***P *trend**				**ns**		

AS = ankylosing spondylitis; CI = confidence interval; MAF = major allele frequency; ns = not significant; OR = odds ratio; SNP = single nucleotide polymorphism.

**Table 2:** Haplotype distribution in patients with AS and controls

Haplotypes rs1264457/rs2074505/rs2074503/rs2269709	Random controls	HLA-B27 controls		*P*	HLA-B27 patients with AS	p1	p2
		Non B*2709 (B*2709) **n = 135**	**n = 40**				
						
AAGA	0.42	0.52	(0.59)*	9 × 10^-4^	0.68	2 × 10^-11^	3 × 10^-4^
GGAG	0.24	0.23	(0.16)	ns	0.12	1 × 10^-4^	0.001
GGGA	0.14	0.10	(0.10)	ns	0.05	7 × 10^-4^	ns
Others	0.20	0.15	(0.15)	ns	0.15	ns	ns

in brackets = haplotype frequencies referring to 40 HLA-B*2709 positive subjects which had been enclosed in the statistical analysis; p1 = HLA-B27 positive patients with AS vs random controls; p2 = HLA-B27 positive patients with AS vs the pool of the HLA-B27 positive controls. AS = ankylosing spondylitis.

## Discussion

The data reported here validate our previous observation of a strong association between a functional polymorphism of the HLA-E gene and AS in Sardinia. It has been shown that the signal peptide of the HLA-B27 molecules binds with low affinity to the HLA-E Arg128 variant [11] and therefore the absence of the alternative variant, Gly128 in patients, could result in a reduced expression of the HLA-E molecules on the cell surface. This, in turn, could lead to an imbalance of the inhibitory function of HLA-E on natural killer (NK) activity or to an alteration of regulatory functions as it has been shown in animal models of autoimmune diseases [12]. More and more reports are involving the HLA-E-NKG2A pathway in autoimmune/inflammatory diseases. Recently, a polymorphism mapping in the HLA-E gene has been found to be associated with Kawasaki disease [13] and the HLA-E-NKG2A pathway is likely to play a role in celiac disease [14]. From the data reported here, it seems unlikely that a HLA-E neighboring gene rather than HLA-E itself, could be the real factor contributing to the disease. The data reported here strongly suggest that NK activity could be involved in the pathogenesis of AS [15] although the HLA-E molecules themselves are not necessarily responsible for this in all populations. As an example, two studies performed in a Spanish and an Asian population reports an imbalance of KIR alleles between patients with AS and HLA-B27-matched controls but these data have not been replicated in a further study in an English cohort [16-18]. This might indicate that different pathways have been selected in different populations based on their genetic background and environmental pressure, leading to the impairment of a crucial function, which can be imbalanced towards an exceeding effector or a defective regulatory function. If this is the case, the more promiscuous the population is, the more difficult it can be to identify some of the minor predisposing genes. For this reason, isolated populations which have undergone strong selection by environmental factors as it occurred in Sardinia, might be particularly informative to single out genes that predispose to complex diseases particularly in those cases in which a gene with a strong effect, such as HLA-B27, plays a leading role [19].

## Conclusions

There is a strong association between a gene mapping in the region between HLA-E and HLA-C and AS in Sardinia. This gene is most likely to be the HLA-E itself because the functional polymorphism rs1264457 encoding for an Arg at position 128 found to be functionally relevant shows the strongest association.

## Abbreviations

ABCF1: ATP-binding cassette, subfamily F (GCN20), member 1; AS: ankylosing spondylitis; ERAP1: endoplasmic reticulum aminopeptidase 1; FOR: Forward; GNL1: guanine nucleotide binding protein-like 1; HLA: human leukocyte antigen; IL: interleukin; NK: natural killer; PCR: polymerase chain reaction; PRR3: proline-rich polypeptide 3; RANP1: member RAS oncogene family pseudogene 1; REV: Reverse; SAP: shrimp alkaline phosphatase; SEQ: Sequencing; SNP: single nucleotide polymorphism.

## Competing interests

The authors declare that they have no competing interests.

## Authors' contributions

FP, FB and EC have performed DNA extraction, genotyping and statistical analysis. AC, AV and AM have contributed to the recruitment, analysis and diagnosis of the patients. CC has contributed his collection of HLA-B27-positive controls and their HLA-typing in Sardinia. MTF, IC, CC, AM and RS have contributed to the design of the study, and to the writing and revision of the manuscript.
